# Congenital Adrenal Hyperplasia: Review from a Surgeon’s Perspective in the Beginning of the Twenty-First Century

**DOI:** 10.3389/fped.2013.00050

**Published:** 2014-01-02

**Authors:** Lisandro Ariel Piaggio

**Affiliations:** ^1^Universidad Nacional Del Sur, Abordaje Quirúrgico de las Enfermedades, Cirugía y Urologia Infantil, Bahía Blanca, Argentina; ^2^Hospital IGA Dr. J. Penna, Pediatría, Cirugía Infantil, Bahía Blanca, Argentina

**Keywords:** congenital adrenal hyperplasia, vaginoplasty, clitoridoplasty, sexual functioning, long-term follow-up, sex assignment, sex of rearing, 21-hydroxylase deficiency

## Abstract

Congenital adrenal hyperplasia (CAH) most commonly due to 21-hydroxylase deficiency is the most common type of disorder of sex development. This review will focus on CAH addressing historical and current surgical techniques with their anatomical foundations, with special attention to long-term results and outcomes on sexual function, patient satisfaction, patient attitude toward surgery, and ongoing controversies in management of these patients.

## Introduction

The last century has witnessed from the description of the DNA, the basis of genetic information, to the entire sequencing of the human genome (1). Huge advances in all areas of science and technology have revolutionized humanity and the way we live, think, and work. Medicine evolves continuously and as a result the way we treat patients. Every new advance offers new possibilities to patients as well as new dilemmas and responsibilities. Genital ambiguity continues to challenge care givers and thanks to public access to information the body of knowledge is changing continuously creating new controversies.

Ambiguous genitalia have always kept the attention of humanity. Along history of human kind the image of hermaphroditism has been seen from abhorrent to mythologically divine (2).

Changing attitudes toward this problem has given origin to new nomenclature. The term “disorders of sex development” or DSD, as less pejorative than intersex is nowadays universally accepted. The Chicago consensus statement on management of these disorders has defined the nomenclature and probably marks a milestone to a higher standard of care (3).

### Historical aspects and evolution of surgical technique

Treatment with cortisol replacement became available in the 1950s. Since then survival for salt wasting congenital adrenal hyperplasia (CAH) was possible and surgical correction become an issue. The initial goals of surgical repair were to improve the cosmetic appearance of the genitalia and make the vagina accessible to the perineum. Surgical techniques evolved with time to make not only “cosmetically accepted” genitalia but also normally functioning (3). Current surgical techniques were rationally modified based on new anatomical and functional knowledge. The European consensus statement on 21-Hydroxylase Deficiency (21OHD) published in 2002 states the goals of surgery are: (i) genital appearance compatible with gender; (ii) unobstructed urinary emptying without incontinence or infections; (iii) good adult sexual and reproductive function (4).

Fortunoff and Lattimer described and inverted *U*-flap vaginoplasty in 1964 with the principle of making the vagina open to the perineum with a skin flap (5). The technique was based on skin mobilization to reach the vagina rather than vaginal dissection to reach the perineum. Because of its simplicity became accepted worldwide and continues to be used with modifications 49 years after its introduction for low confluence vagina (6). A different concept was introduced by Hendren and Crawford in (7) for a high confluence vagina, the “pull through” procedure. They advocated that with this technique a high vagina, not suitable for a cutback procedure could be exteriorized to the perineum without disturbing the urethral sphincter. They proposed surgery before 1 year of life. In this paper they stated that clitorectomy was virtually always desirable since it offered the best cosmetic result and there was evidence it did not adversely affect sexual function in adult life. They presented seven cases with a “satisfactory result.” The results were based on the ability to bring a perineal vagina. Time of follow-up was not reported and presumably was short. There was no information on vaginal stenosis, sexual function, or urinary outcome but they concluded that the patients should be able to have “satisfactory intercourse in later life” (7).

In the last five decades vast information in clitoral function became available and several reports specifically have addressed clitoral anatomy, neuroanatomy and embryology, clitoral innervation and its development (8–16). Today, reduction clitoroplasty, where the glans is preserved and part of the erectile bodies are excised, is the most widely accepted and used technique (3, 4, 17). Clitorectomy has not been considered a reasonable option for decades.

## Surgical Management of CAH

Most surgeons would nowadays prefer to perform a one stage repair with clitoral reduction, vaginoplasty, and labiaplasty/introitoplasty at early age. This is the author’s preference. In a recent specialist survey 78% of respondents preferred early surgery, before the age of 2 years as the preferred strategy for feminizing genitoplasty in patients with CAH. The survey infers that skin flap is the most popular technique for vaginoplasty, followed by *en-block* urogenital mobilization (most surgeons use more than one technique) (6) The consensus statement ESPE/LWPES CAH working group advocates a one stage repair before 12 months of age for those virilized patients with a high confluences of the urethra and vagina, while discourages surgery between 1 year of life and puberty and in cases where the degree of virilization is less (4). There is increasing evidence that no matter the technique for vaginoplasty used, introital, or vaginal stenosis are quite common needing surgical revision or vaginal dilatations after puberty. For that reason delaying vaginal surgery after puberty (two stage repair) or the whole genitoplasty at a time when the patients can take active participation in decision making should be considered (18–20). Detractors of early surgery base their approach on the concern that there is insufficient evidence that early surgery benefits gender identity, potential impairment in clitoral sensitivity and high rates of revision for vaginoplasties. Despite several publications in this area, there is a lack of evidence to suggest that the late approach can achieve better outcomes.

Two stage repair or delaying operations until puberty or adulthood are currently practiced in many centers and strongly advocated by some authors. For this reason clitoral and vaginal surgery results and psychosexual function will be discussed separately. It is understood this is only for practical purposes. Many surgeries were performed simultaneously, surgical revisions are common and the ultimate outcome is patient satisfaction with its urogenital function and body image. Urinary outcome will not be addressed in this review (21). Even though the London group found that patients with CAH are more likely to have significant urinary symptoms than normal controls and felt this issue is frequently underestimated when evaluating patients affected with CAH (22), there are recent well designed studies that do not confirm this findings (23–26). The reviewed literature in this pathology is more consistent with long-term genital and sexual problems than with urinary tract symptoms; the former affecting quality of life significantly more the than the later.

### Clitoral, urethral and vaginal anatomy, and rationality for surgical changes

The clitoris is a multiplanar structure with a broad attachment to the pubic arch. Its components include the erectile bodies, paired bulbs, and paired corpora, which are continuous with the crura. The clitoral neurovascular bundles (paired terminations of the pudendal neurovascular bundles) ascend along the ischiopubic rami to meet each other and pass along the superior surface of the clitoral body. The neural trunks pass largely intact into the glans. These nerves are at least 2 mm in diameter even in infancy. Large bundles of nerves course along the corporeal bodies with the highest density on the dorsal aspect or top. No nerves are found at the 12 o’clock position. The evoked and spontaneous clitoral electromyography activity seems to indicate a sympathetic tonus of the corpus clitoris. The glans clitoris is an anatomically separate structure capping the end of the corporeal bodies which are well defined by the tunica albuginea. Glans innervation is provided by multiple perforating branches entering at the dorsal junction of the corporeal body and glans. Its histological immunoreactivity is consistent with sensory nerves. The lowest density of nerves in the glans is on the ventral aspect (8, 9, 13, 15).

The distal urethra and vagina are intimately related structures, although they are not erectile in character. They form a tissue cluster with the clitoris. This cluster appears to be the locus of female sexual function and orgasm. The perineal urethra is embedded in the anterior vaginal wall and is surrounded by erectile tissue in all directions except posteriorly where it relates to the vaginal wall (11). The bulbs of the vestibule are inappropriately named as they directly relate to the other clitoral components and the urethra (9). The anterior and lateral vaginal walls are the areas most densely innervated by nitric oxide immunoreactive nerves (autonomic nerves). The innervation of the urethral sphincter complex is provided by the vaginal nervous plexus originating from S2 to 4 and by the intrapelvic branch of the pudendal nerve (10). Clitoral innervation extends from S1 to L2 with no labeling seen in the L3 spinal cord (14). In order to better preserve genital innervations and improve surgical outcome in urinary continence and sexual function, the distal urethra and vagina should not be separated and dissection of the bladder from the uterus below the bladder neck should be avoided. According to the current knowledge, the pull through technique for a high confluence vagina is outdated and other modern techniques should be considered for these cases.

According to their studies Baskin et al. proposed a new surgical technique with the aim of preserving the majority of the clitoral neurovascular bundles and nerves (8). Since then there have been some modifications on surgical technique (e.g., glands reduction is no longer recommended by the majority of authors), but its principle remains the standard of care. Poppas et al. demonstrated that nerve sparing ventral clitoroplasty preserves dorsal nerves by histological examination of excised erectile tissue from patients with CAH at the time of genitoplasty (27). Techniques that base the survival of the clitoral cup on the ventral aspect of the clitoris should be discouraged since may result in glans necrosis (28).

The consensus statement on management of intersex disorders recommends that the surgical procedure should be anatomically based to preserve erectile function and the innervation of the clitoris since orgasmic function and erectile sensation may be disturbed by clitoral surgery (3). Based on the mentioned publications some other recommendations can be made (Table 1), but reports on the long-term results with emphasis on functional outcome rather than a strictly cosmetic appearance are encouraged. We should keep in mind that preservation of the anatomical structures is basic for a good functional result, but assuming there is good function because the anatomy appears preserved is a fallacy.

**Table 1:** **Current surgical recommendations on feminizing genitoplasty and clitoral surgery**.

The attachment of the clitoris to the pubic arch should not be disrupted
Dorsal neurovascular bundles should be preserved. There may be significant lateral branches along the corporal tissue
The neurovascular bundles branch distally in a fan fashion toward the glands clitoris
Reduction of the clitoral erectile tissue is better achieved with a ventral incision along the urethral plate
The urethra and vagina should not be separated
Dissection of the bladder from the uterus below the bladder neck should be avoided
Using labial and clitoral skin to cover the new introitus is more physiological than rotational skin flaps from the buttocks and perineum

### Reports on clitoral surgery and sexual outcome

Minto et al. questions the ability of modern operations to achieve better functional results than the old fashion ones. In their study they recruited 39 adult women with DSD, 28 had been sexually active of whom 18 had clitoral surgery and 10 did not. Clitorectomy was the most common operation, but some have reduction clitoroplasty with nerve bundles preservation. More than one clitoral operation was performed on 14% of participants. There were difficulties in sexual function in both the surgery and no surgery groups. However, the surgery group had a significantly higher rate of orgasmic difficulties and lack of sensuality than those who had not had surgery. Seven (39%) of 18 who had had clitoral surgery answered that they always found it impossible to have an orgasm compared with none of those who had not had clitoral surgery (*p* = 0.03) (29). Crouch and colleagues from the same institution further reported on six adult patients with CAH who had feminizing genitoplasty. They found that five patients had abnormal results for sensation of warmth to the clitoris, and all had abnormal results for sensation of cold. Two patients had no identifiable glands or clitoral tissue, with an appearance consistent with total clitorectomy, although the original operation was noted as clitoral reduction in all patients. The five sexually active women who completed a validated questionnaire reported sexual difficulties, particularly in the areas of infrequency of intercourse and anorgasmia (30). Alizai reported unsatisfactory anatomical results in 46% of their patients because of absent clitoris, atrophied clitoris, small residual glands, or unacceptably prominent glands. Functional outcome or parent/patients satisfaction with surgery was not assessed (31).

Similarly to Crouch, Lesma reported clitoral sensitivity, both thermal and vibratory, was significantly decreased in all patients compared with healthy controls (*p* < 0.01), on adult women following Passerini-Glazel feminizing genitoplasty at young age. Surprisingly there was no difference neither in vaginal sensitivity nor sexual function. The same number of patient and controls (91.6%) described stable satisfactory relationship with active sexual desire, good arousal, adequate lubrication, and orgasm (32).

It is likely these studies may not completely reflect the outcome of modern surgical techniques, since others have reported better outcome, but it is clear that an operation could be worse than no surgery. Reduction clitoroplasty may impair clitoral sensitivity or viability jeopardizing future sexual outcome, but how much this will influence adult life is controversial. Further reports on long-term results of clitoral surgery addressing sexual outcome are in need.

Gearhart et al. demonstrated that terminal latencies electromyography before and after phallic debulking were no significantly changed after feminizing genitoplasty. While it was proven nerve conduction was no altered in 5/6; sensation was not tested (33). Chase from the Intersex Society of North America replied than a patient treated in 1975 with the same surgical technique the authors proposed considers herself to have undergone clitorectomy and her sexual function as being destroyed because orgasm is so difficult to reach and so rarely attained, despite not having lack of sensation (34) This underlines preserving the anatomy may not be enough for good sexual function. On the other hand there are anorgasmic women which were born with normal genitalia and never had genital surgery. In a study from Sweden, there were no abnormalities in the appearance or size of the reconstructed/reduced clitoris in any patient following feminizing genitoplasty at early age. There was no difference in vibration and light touch/deep pressure sensitivity between patients treated with single clitoridal reduction compared to a group of normal healthy women – except for the one operated twice. All operated women considered the anatomy of their outer genital region normal and they would recommend the operation to other affected women with CAH (35). Similarly Yang and colleagues reported viability of the clitoris in all 49 patients following surgery, assessed by capillary perfusion. Clitoral sensitivity was assess with two methods in 9 and 10 patients and was well preserved in all. Furthermore in two patients the test showed no changes before and after surgery. The drawback of this study is that these tests were not validated in healthy women (36).

In summary there is controversy on functional outcome of clitoral surgery despite using modern techniques. Reduction clitoroplasty may alter clitoral sensitivity and preserved innervations/nerve conduction does not necessarily assure well sexual function. On the contrary, despite genital sensation been altered by surgery, sexual outcome, and patient satisfaction may not be impact.

### Reports on vaginal surgery, sexual outcome, and surgical trends

In assessing the long-term follow-up of 16 women (of an original 32 children), Lattimer’s group concluded in 1976 that the results of vaginoplasty were so poor that the operation should not be done before puberty [cited in Ref. (20)]. Bailez reported on long-term results of Fortunoff vaginoplasty for CAH. Revision rate in these series of 28 patients was 78% to achieve a normal vaginal outlet with a initial success rate of 72% for the further repair (37). Alizai et al. reported that all 13 patients who underwent feminizing genitoplasty in infancy who were examined under anesthesia would require surgical revision of the vagina for normal comfortable intercourse (12 flap vaginoplasty, 1 pull through) (31). The London group assessed 44 adolescents (21 CAH) who had had surgery in early childhood. With a mean follow-up of 13.2 years, 98% would require further treatment to permit penetrative intercourse. Although 23% of subjects only required dilatation therapy to the vagina, 77% were in need of further surgery. The report stressed that genital ambiguity cannot be corrected in infancy by a single procedure since the majority of patients will be in need for revision vaginoplasty (19). In other series, six of the seven patients with an initial single-stage procedure required re-vaginoplasty in puberty (38). Krege reported the long-term follow-up of 25 patients with CAH who underwent vaginoplasty (24 Forunoff flaps, 1 pull through). Intravaginal stenosis was found in a significantly lower proportion of patients (36%) with the cosmesis of the external genitalia found to be normal from the examiner’s perspective in all (39). Similarly Sircili reported 33% of stenosis on 12 patients who went into puberty after feminizing genitoplasty, of whom the majority (3/4) were managed with dilatations, and only 1 requiring surgical revision. Three patients are sexually active, one presented pain with intercourse (28).

The vaginoplasty with the bilateral labioscrotal flap presented by Moriya et al. seems to have no advantage presenting stenosis or stricture in 39 and 15%, respectively and the need for surgical revision in at least 30% of their patients on median follow-up (40).

The Passerini-Glazel Feminizing Genitoplasty offers a mucosal introitus which usually doesn’t present stenosis, but the suture line within the native vagina and the inverted urogenital sinus frequently does. In the report from Lesma with 17 years of experience, they presented 20 females that had gone through menarche. No patients indicated problems with pruritus, leukorrhea, or malodorous vaginal discharge. Suture line with mild stenosis treated with Hegar dilatation was present in 21.7%, while 34.8% presented severe stenosis requiring surgical revision (YV plasty) (41). No information was provided on revision vaginoplasty, but they recently presented extended follow-up in 12 women on clitoral sensitivity and sexual outcome with good results compared to a group of healthy patients (see above clitoral surgery) (32).

Aguirre reported that 5/6 (83%) of their patients who underwent one stage vaginoplasty presented stenosis, while none (0/7) in those who underwent delayed vaginoplasty at 9–13 years of age, changing they current surgical approach to a two stage repair for high confluence vagina (18). A Swedish group reported on eight women who had differed vaginoplasty between 10 and 16 years of age (all have clitorplasty at age 0–3 years) using perineal skin flaps (8) or skin grafts (1). All women reported that the vaginal reconstruction was successful as far as sexual activity was concerned and six of the eight had stable relationships with boyfriends or husbands. The gynecological examination showed adequately positioned vaginal reconstructions with good wound healing and no constricting scar tissue (35). The Leipzig experience with two stage vaginoplasty seems excellent as far as vaginal patency is concerned. Of the 58 patients (41 CAH), 32 (55%) had already achieved sexual intercourse (Mean age at first sexual intercourse 18.2 years) and in 17 the postoperative result would allow sexual intercourse, while in 3 the possibility of sexual intercourse is uncertain (3 refused surgery, 1 lost to follow-up) (42).

In summary most patients with CAH operated with a Fortunoff flap vaginoplasty performed at young age will need future minor or major surgical revision. Other techniques like Passerini-Glazel Genitoplasty or labioscrotal flaps developed frequent stenosis but, severe stenosis needing surgical revision is less common (about a third of patients). Reports on En-block urogenital mobilization with the use of urogenital or skin flaps are promising, with excellent short term and medium results, but extended follow-up is currently unavailable (43–47) Two stage feminizing genitoplasty, delaying vaginal reconstruction until or after puberty seems to have excellent functional outcome.

### Psychosexual outcome

Studies addressing specifically this issue are scant, but despite the frequent revision surgery rate on vaginoplasty, and the apparent disruption on clitoral sensation (30), women with CAH adapt well to their chronic pathology, and psychosexual outcome appears more positive in few recent reports than the worrisome previous results (32, 38).

In a comparative study performed by May and colleagues in (48) found that patients with CAH were less sexually experienced and expressed a lower level of sexual interest than a group of adult diabetic women. Also, the women with CAH had specific sexual function difficulties with penetration, pain during intercourse, and orgasm (48). But when comparing role-play and feelings of self-esteem, Mosely et al. found that juvenile onset diabetics and CAH women the differences are very small (49).

Wisniewski studied the long-term satisfaction with gender, cosmetic appearance and function of the genitalia, and surgical management practices on patients with CAH comparing simple virilizing form, salt wasting and a normal control group. Of note 63% of the patients underwent clitorectomy. When assessing surgical outcome physician rated appearance of the external genitalia better than patients did. CAH salt loosing patients reported significantly greater sexual concerns, poorer genital function and fewer sexual relations with partners than their counterparts with the simple virilized form or control women. Both groups reported moderate satisfaction with the adult appearance. Women opinion on optimal timing for feminizing genitoplasty was 23% at adolescence or adulthood and 77% before that age (48% at infancy) (50).

Homosexuality may be more prevalent in women affected with CAH than in the general population, but most reports were sexual preference was assed show that women considered themselves heterosexual. Nevertheless female identity may be question. This was found in 37% of women in Wisniewski study (none desired to change sex to male), 2/8 (25%) of Frost-Arner series, and 4/12 (33%) with occasional to chronic doubts in Vates report (35, 50, 51). Zucker found that 50 out of 53 adult women with CAH were happy with a female gender identity (52).

Women with CAH usually have their first sexual intercourse at an older age than that reported from control groups (19, 29, 30, 32, 39). Kuhnle et al. (53) studied 45 women with CAH and compared them with 46 controls, finding that women with CAH had social and sexual milestones later than age-matched controls, but showed no greater preference for same-sex relationships [cited in Ref. (54)]. Krege reported that only 6 of 15 women with CAH at an age when most women within the general population have sexual intercourse (17–32 years old) were sexually active or had had sexual intercourse. Although the majority of women were capable of orgasm with clitoral or vaginal simulation, many avoid sexual activity because of doubts about the appearance of their external genitalia and their normal vaginal function, and the subsequent anxiety (39).

Stikkelbroeck and colleagues reported on long-term outcome of feminizing genital surgery. They found no psychopathology with the YASR test, except for one patient with a slightly elevated externalizing score (38). Sexual developmental milestones (romantic interest, falling in love, kissing and petting, coitus) had been reached by all, except for 1 patient who did not have coitus yet. In the patient group, satisfaction with height, body hair, and external genitalia and sexual fantasies and interest, was not different compared to the control group, except for satisfaction with total body appearance, which was significantly lower in the patients. In the interview, two patients called themselves bisexual, the other four heterosexual. None of the patients had homosexual contacts (38).

### Toward a new standard

It is generally felt that surgery that is carried out for cosmetic reasons in the first year of life relieves parental distress and improves attachment between the child and the parents The systematic evidence for this belief is lacking. Despite what we, as physicians, might consider a good or poor cosmetic and even functional outcome, patients have expressed both dissatisfaction and satisfaction, respectively. Perhaps we are asking the wrong questions. Our ultimate goal, as physicians and surgeons, is to satisfy patient expectations. Establishing what creates a good sexual QoL for intersex patients is the very essence of knowledge that would be most valuable and important to us (55).

Despite the efforts in improving surgical techniques there is no clear evidence that our current intervention will aid in a better sexual function in adult life. Is not only what the surgery does to the body, is what the conjunction of the treating team in a given socio-cultural environment does to a person. Psychosexual function is far the way up than a subjective good surgical result.

It is sensitive to avoid patient stigmatization, be clear with surgical expectations, avoid repeated pelvic examinations when not crucially needed, especially throughout toddler and child development, offer patients and family attention with a multidisciplinary team with special emphasis in psychological support and encourage participation in self-help groups.

## Final Reflexions

Surgical technique for virilized genitalia in CAH has evolved from excisional surgery of the clitoris, the most innervated organ in the human body, to very refined surgery with precise anatomical landmarks to preserve the majority of nerve terminals in reductions clitoroplasty. Several aspects of vaginoplasty and introitoplasty had been developed to improve cosmetic appearance and avoid vaginal and introital stenosis. While most surgeons caring for these patients claim they achieve “good cosmetic results” the long-term functional outcomes are scant and mostly disappointing. There is no doubt the standards have increased and today is not enough to say that a “normal looking” genitalia congruent with rearing sex from early age are enough. We need to know how this will work, if our “almost perfect” surgical technique will have a positive or negatively impact on sexual function and quality of life.

There are concerning reports on these patients about, sex identity doubts, anorgasmia or difficulty with orgasm, avoidance of sexual activity, and low scoring in many aspects of sexual life.

Is no surgery an option? From the surgeon’s perspective is hard to “stay quiet” facing a chromosomally female patient with a treatable endocrinological disease with virilized genitalia feasible of changing to “normal looking female genitalia.” The available data suggests that re-operations for redo clitoral reduction, vaginoplasty, and introital stenosis are quite common and the greater the number of operations, the greater the chance of developing scars and jeopardizing sexual outcome with a denervated or dysfunctional clitoris and painful intercourse through a scarred vagina.

In the best possible scenario one stage genitoplasty at an early age will render in a normal looking and innervated genitalia that will function normally in the future if hormonal substitution and continued medical attention is carried out through-life. The contrary may be true. When counseling parents of these patients we should clearly state that we think rearing kids with sex-congruent looking external genitalia is more reasonable than leaving them untouched until puberty to avoid physiological trauma; but we have no clue if this is right. There is no strong scientific evidence to confirm this statement, neither than the opposite is better.

It is clear than no surgery will be less damaging to any anatomical structure than the best possible operation performed in a center of excellence. But currently we have no choice other than surgery to correct the altered genitalia, no matter the sex of rearing or the age at the first operation. There is a need for continued investigation in the functional implications of surgery that will necessary deal with mobilization of the urogenital structures, with special emphasis on long-term results of modern techniques, timing of surgery, and impact on quality of life. Psychological support should not be underestimated and its impact and timing of implementation should be studied as well.

## Conflict of Interest Statement

The author declares that the research was conducted in the absence of any commercial or financial relationships that could be construed as a potential conflict of interest.
